# Electrical Impedance-Based Characterization of Hepatic Tissue with Early-Stage Fibrosis

**DOI:** 10.3390/bios12020116

**Published:** 2022-02-13

**Authors:** Susana Fuentes-Vélez, Sharmila Fagoonee, Alessandro Sanginario, Marco Pizzi, Fiorella Altruda, Danilo Demarchi

**Affiliations:** 1 Department of Electronics and Telecommunications (DET), Politecnico di Torino, Corso Duca degli Abruzzi, 24, 10129 Turin, Italy; susana.fuentes@polito.it (S.F.-V.); danilo.demarchi@polito.it (D.D.); 2 Institute of Biostructure and Bioimaging (CNR), Molecular Biotechnology Center (MBC), Via Nizza, 52, 10126 Turin, Italy; sharmila.fagoonee@unito.it; 3 Eltek S.p.A, Strada Valenza 5/A, 15033 Casale Monferrato, Italy; m.pizzi@eltekgroup.it; 4 Department of Molecular Biotechnology and Health Sciences, Molecular Biotechnology Center (MBC), University of Turin, Via Nizza, 52, 10126 Turin, Italy; fiorella.altruda@unito.it

**Keywords:** EIS, biopsy, 3D cell culture, bile duct ligation, circuit model

## Abstract

Liver fibrosis is a key pathological precondition for hepatocellular carcinoma in which the severity is confidently correlated with liver cancer. Liver fibrosis, characterized by gradual cell loss and excessive extracellular matrix deposition, can be reverted if detected at the early stage. The gold standard for staging and diagnosis of liver fibrosis is undoubtedly biopsy. However, this technique needs careful sample preparation and expert analysis. In the present work, an ex vivo, minimally destructive, label-free characterization of liver biopsies is presented. Through a custom-made experimental setup, liver biopsies of bile-duct-ligated and sham-operated mice were measured at 8, 15, and 21 days after the procedure. Changes in impedance were observed with the progression of fibrosis, and through data fitting, tissue biopsies were approximated to an equivalent RC circuit model. The model was validated by means of 3D hepatic cell culture measurement, in which the capacitive part of impedance was proportionally associated with cell number and the resistive one was proportionally associated with the extracellular matrix. While the sham-operated samples presented a decrease in resistance with time, the bile-duct-ligated ones exhibited an increase in this parameter with the evolution of fibrosis. Moreover, since the largest difference in resistance between healthy and fibrotic tissue, of around 2 kΩ, was found at 8 days, this method presents great potential for the study of fibrotic tissue at early stages. Our data point out the great potential of exploiting the proposed needle setup in clinical applications.

## 1. Introduction

Approximately 2 million deaths occur every year worldwide due to severe liver diseases, imposing a significant burden on healthcare systems [1]. Liver diseases have been estimated to be the fifth most common cause of death worldwide, with a rising trend [2]. Liver fibrosis surges as a wound-healing response to chronic injuries. An imbalance in the synthesis and degradation of the extracellular matrix (ECM) alters the hepatic tissue architecture, in which scar tissue replaces liver parenchyma [3]. It is known that the degree of liver fibrosis has an effect on the management and prognosis of chronic hepatitis; therefore, accurate staging of liver fibrosis is essential in evaluating its evolution to cirrhosis and associated complications such as hepatocellular carcinoma and liver failure. Importantly, the earlier liver fibrosis is diagnosed, the faster the causal agent can be removed, thus resulting in early liver fibrosis reversibility [3].

Liver biopsy remains the gold standard for evaluating fibrosis using histologically standardized, universally accepted scoring systems [4]. However, histological preparation and analysis are time-consuming and pathologist expertise is required. Thus, label-free, real-time diagnostic tools are urgently required. Real-time histopathology is a promising approach but requires sophisticated and expensive instruments [5]. Noninvasive in vivo bioimpedance measurements have been introduced as an innovative approach for the detection of liver fibrosis [6]. Morgan et al. described the potential of impedance-based techniques in hepatology research and have identified the main platforms used for impedance-based cell analysis with hepatocytes in monolayer cultures in vitro. The application of these techniques in vitro and ex vivo to 3D structures still needs to be addressed and would be an important breakthrough [7]. Previous work on the electrical characterization of hepatic tissue has focused on cancerous tissue [8] or liver steatosis [9,10], while liver fibrosis has been less explored.

Impedance-based measurements have gained tremendous interest lately due to their non-invasiveness and label-free recordings. We recently showed that drug-induced chemoresistance development in colon cancer cells can be determined by long-term, impedance-based, cell culture monitoring [11]. In the present work, we further analyzed the electrical signature of healthy and fibrotic hepatic tissue through non-destructive, label-free impedance analysis of biopsies to determine whether this approach could assist in detecting early fibrogenesis. Electrochemical impedance spectroscopy (EIS)-based ex vivo analysis was performed, and changes in the tissue, in the form of a dielectric response, were recorded to explore its diagnostic potential. The well-characterized obstructive cholestasis-induced liver fibrosis model (Bile Duct Ligation, BDL) was used for this purpose. BDL induces a series of events, including acute hepatobiliary injury; proliferative response of parenchymal and non-parenchymal liver cells; and up-regulation of pro-inflammatory and pro-fibrotic cytokines and metabolic enzymes, which lead to increased deposition of ECM and scar tissue formation [12,13,14]. Liver fibrosis is histologically detected starting from 7 days after surgery [13,15].

EIS data obtained at 8, 15, and 21 days post-surgery and fitted for the computation of an equivalent circuit model indicate, to our knowledge, for the first time that biological tissue characterization, through the present approach, might be exploited in a biopsy needle setup such as the one described by Park et al. for real-time EIS measurement during the biopsy process [16].

## 2. Materials and Methods

### 2.1. Hepatic Fibrotic Model, Biopsies Preparation, and Histology

**Figure A1:**
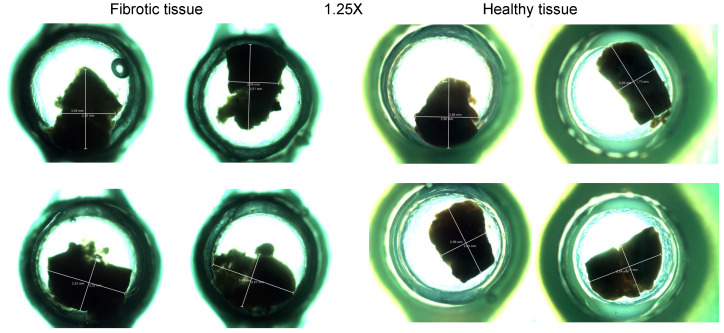
Microscopy images of tissue biopsies inside culture chambers. Images were taken under a Olympus BX41 microscope at 1.25×.

BDL and sham surgical procedures were performed as previously described in [13]. Briefly, mice were subjected to double ligation of the common bile duct without dissection of the duct between the ligatures. Sham-operated mice underwent the same surgical procedures as BDL mice but without ligation. All experiments were performed in accordance with the Italian legislation on the protection of animals (Protocol number: CC652.109) and the University of Turin Guidelines. Mice were sacrificed at 8, 15, and 21 days post-surgery, and formalin-fixed, paraffin-included liver sections were stained with hematoxylin/eosin (H/E) and PicroSirius red (PSR). Liver lobes were taken at the indicated time points, and ex vivo biopsies weighing approximately 13 mg and measuring 3 mm in diameter and 2 mm in height were dissected with a catheter punch and used in the experiments described below (Figure A1, Table A1, Table A2 and Table A3).

### 2.2. EIS Measurement Setup and Data Acquisition

**Figure 1:**
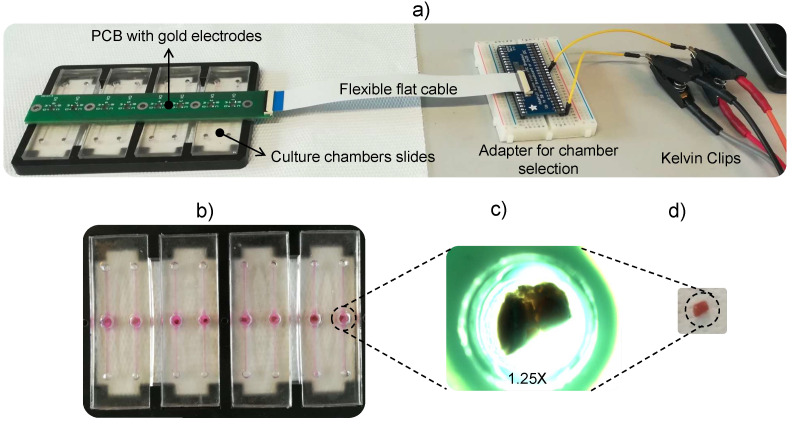
Experimental setup: (**a**) Four culture chamber slides with two chambers each were fabricated in a biocompatible polymer. A PCB held the couple of vertical gold electrodes for each chamber. A flexible flat cable connected the PCB to an external adapter that allowed for manual selection of the culture chamber in which the measurement was performed. Kelvin Clips were connected to the 4192A LF Impedance Analyzer (Keysight Technologies). (**b**) Top view of the culture chambers slides with biopsies. (**c**) Microscopy image of a biopsy suspended in culture medium inside the culture chamber (1.25×). (**d**) Non-suspended biopsy outside the culture chamber (geometry obtained with catheter punch).

Impedance measurements were performed with a 4192A LF Impedance Analyzer (Keysight Technologies, Santa Rosa, CA, USA) through a custom-made graphical user interface (GUI) developed in LabVIEW™ (National Instruments, Austin, TX, USA). The GUI allowed for parameter tuning and automatic data recording. As can be observed in Figure 1, the system was designed with eight chambers. Culture chambers were filled with 150 μL of William’s E Medium, GlutaMAX Supplement (Gibco, Thermofisher Scientific, Waltham, MA, USA), and the volume was kept constant for the entire measurement, under static fluidic conditions. The eight samples were prepared, and measurements were performed sequentially, processing one chamber at a time. Impedance module (|Z|) and phase (θ) were measured with two electrodes using Kelvin Clips, with an excitation signal of 50 mV in the frequency range between 500 Hz and 1 MHz, through a logarithmic sweep. The setup consisted of cylindrical gold-plated electrodes welded on a printed circuit board (PCB) and inserted vertically from the top of the culture chamber. Culture chambers (5.4 mm in diameter and 5.8 mm in height) were fabricated through a replica molding process using a biocompatible polymer.

**Figure A2:**
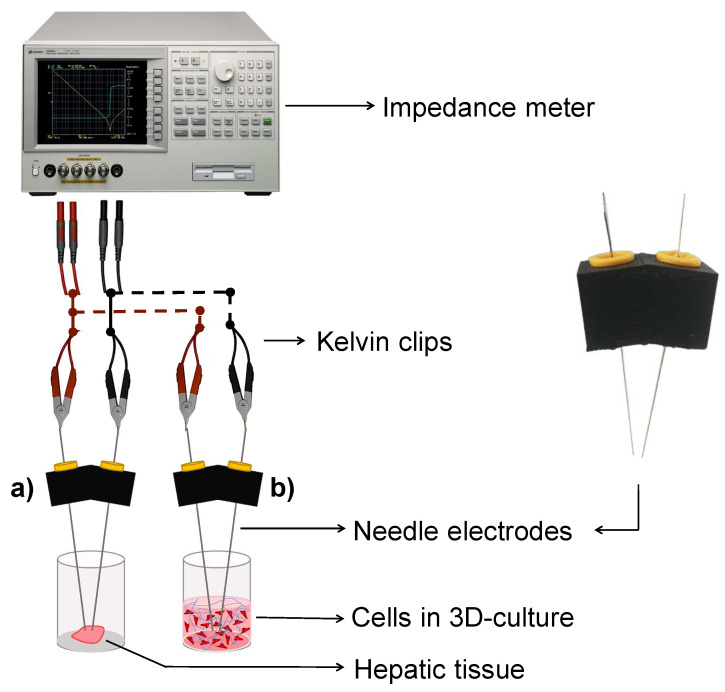
Illustration of the needle electrode setup used for impedance measurements on (**a**) biopsies directly in the absence of culture medium, and (**b**) the 3D cell culture model with Hep-3B cells and type I collagen gel.

Direct impedance measurements were also performed using needle electrodes inserted directly into the tissue with the goal of obtaining its impedance without the contribution of the surrounding culture medium (Figure A2a). The measurement parameters described above were employed.

### 2.3. 3D Cell Culture Model

To dissect the tissue components responsible for the changes in impedance in the fibrotic liver, needle electrodes were employed to perform impedance measurements in 3D cell cultures (Figure A2b). Collagen-sandwiched 3D-culture of hepatocytes were prepared as in [17] with the Hep-3B cell line and type I collagen from a rat tail (Merck). Cells were grown at 37 ${}^{{}^{\circ}}$C, 5% CO${}_{2}$, and 95% air in DMEM (Dulbecco’s Modified Eagle Medium) with GlutaMAX (Gibco by Life Technologies), supplemented with 10% heat-inactivated fetal bovine serum (Gibco by Life Technologies) and 1% Penicillin-Streptomycin (Gibco by Life Technologies). Briefly, a first layer of collagen gel at the final concentration of 2 mg/mL was prepared inside a 96-well plate and left overnight to solidify at 37 ${}^{{}^{\circ}}$C and 5% CO${}_{2}$, followed by rehydration before cell seeding. The second layer of collagen was laid on the cells 3 h after seeding. Three different conditions were studied: no cells, $5\times 10^{5}$ cells/mL, and $1\times 10^{5}$ cells/mL. For each condition, measurements were performed in two wells. Measurements were performed 24 h after cell seeding.

### 2.4. Data Processing and Statistical Analysis

The electrical components of the biopsy immersed in culture medium were measured with respect to the culture medium only for normalization. Furthermore, to reduce the measurement error induced by sample dimensions, mass normalization was performed. The measurements were performed in triplicates; livers from three mice were taken, and from each resected tissue portion, three independent biopsies were obtained for analysis. Data post-processing was performed in MATLAB R2018b^®^.

Data post-processing was conducted as follows:

1. The impedance module of each chamber was normalized to the culture medium measurement and the sample mass (m). The normalized impedance (*NI*) magnitude was ${\left[NI\right]}_{i}=g^{-1}$.
  (1) $$NI_{i}=\frac{|Z_{culture\;medium+sample}{|}_{i}}{|Z_{culture\;medium}{|}_{i}}*\frac{1}{m_{i}}$$
2. Mean curves were obtained from computed *NI* triplicate biopsies from the same mouse liver (*k* = 3, number of biopsy replicates from the same mouse).
  (2) $$\bar{NI}=\sum_{n=1}^{k}\frac{NI_{i}}{k}$$
3. Mean curves were obtained from mice triplicates (*n* = 3, number of mice).
  (3) $$\bar{NI_{f}}=\sum_{n=1}^{n}\frac{\bar{NI}}{n}$$

Mean impedance magnitude (|Z|), normalized only by mass, and impedance phase (θ) were used to fit the equivalent circuit model. Raw data from the 3D cell culture were used, as well, for the equivalent circuit calculation.

## 3. Results

### 3.1. Dielectric Response of Healthy and Fibrotic Tissue Biopsies

**Figure 2:**
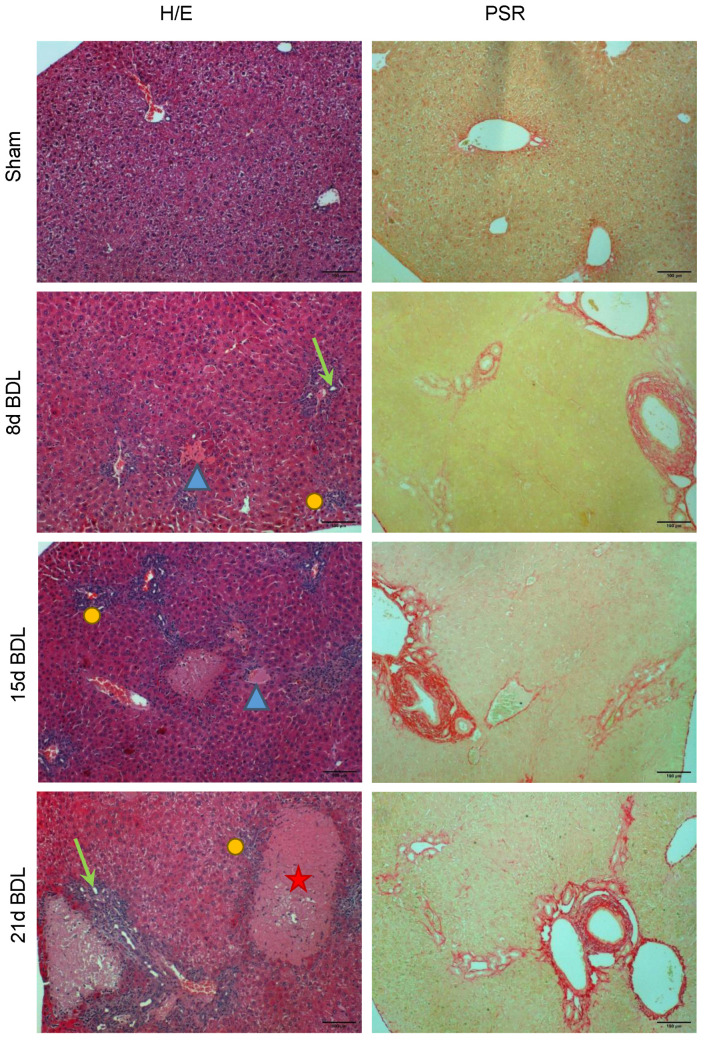
The typical appearance of liver tissue at representative time points after BDL with respect to sham controls is shown. Hematoxylin and eosin (H/E) and PicroSirius Red (PSR) staining reveal the tissue architecture and deposition of extracellular matrix (in red), respectively. The star shows necrotic areas, the arrowheads show bile infarct areas, the arrows show the artificial bile ductules formed, and the dots show inflammatory cell infiltrates. Images were taken at 10× magnification. Scale bar: 100 μm.

The PSR-based histological evaluation of liver sections of mice subjected to BDL revealed a time-dependent increase in ECM deposition around the periportal area starting from day 8 post-surgery with respect to the sham control. Liver fibrosis gradually increased in the BDL mice up to 21 days post-surgery (Figure 2). Liver injury was evident from the H/E stained sections with bile infarcts areas, massive necrosis (corresponding to clusters of injured hepatocytes), the formation of artificial bile ductules (ductular reaction), as well as inflammatory cell infiltrates, resulting from the obstruction of the common bile duct compared with sham controls.

**Figure 3:**
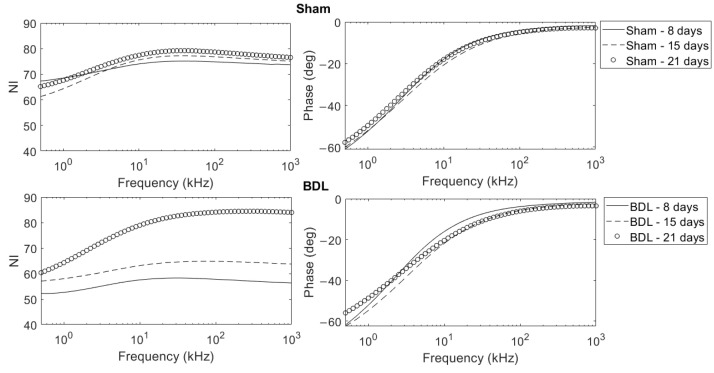
Normalized impedance magnitude and phase curves of sham-operated and BDL liver samples in the range between 500Hz and 1MHz. For each condition (Sham and BDL at 8, 15 and 21 days), three livers were studied. From each liver, three different biopsies were obtained for analysis in triplicates. Therefore, each of the illustrated curves corresponds to the mean value of nine measurements (three mice, each in triplicate).

The normalized impedance magnitude and phase for both sham and BDL liver biopsies are presented in Figure 3. As mentioned in Section 2.4, for each type and condition (8, 15 and 21 days), measurements were performed in triplicates (three biopsies from each liver) and repeated for three mice. Each of the illustrated curves in Figure 3 correspond to the mean value (*n* = 3 mice, 9 measurements in total). A gradual increase in impedance magnitude was observed with fibrosis progression with time, while little variation was observed in healthy tissue. The impedance magnitude of sham-operated tissue was higher than its fibrotic counterpart at 8 and 15 days after surgery. At a late fibrotic stage (21 days post-BDL), a significant increase in impedance was observed, reaching values of the respective sham-operated curve. Little variation was observed in the phase. For further validation purposes, direct tissue measurement was performed by inserting the needle electrodes in the liver biopsy at the intermediate stage of 15 days. The impedance magnitude of the fibrotic tissue was lower than that of the the sham-operated liver, confirming the results initially obtained with this approach (Figure A4).

### 3.2. Equivalent Circuit Model and BDL Evolution Analysis Through Impedance

**Figure 4:**
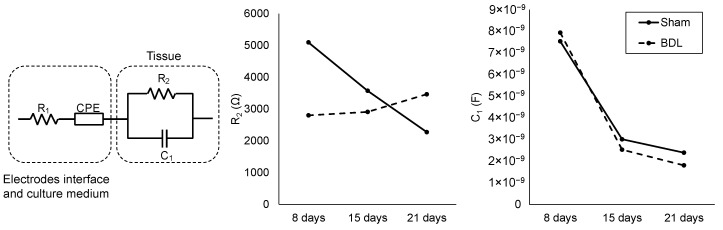
Complete equivalent circuit model, and resistance ($R_{2}$) and capacitance ($C_{1}$) values of the tissue RC model for BDL and sham-operated samples at the three studied time points.

Several models have been proposed related to bioimpedance analysis. In the present work, through impedance analysis, the characterization of fibrotic liver is presented, in which tissue was approximated to a single-dispersion RC model ($R_{2}$ and $C_{1}$ in Figure 4) [18]. The electrode interface and surrounding culture medium were modeled with a series of resistors and constant phase elements ($R_{1}$ and CPE in Figure 4). The mean values for $R_{1}$ and CPE, obtained through the data-fitting procedure, are 24.5 kΩ and 13.2 Fs${}^{a-1}$ (*a* = 0.83), respectively. In electrochemistry, CPEs are often used to represent the double-layer capacitance between electrodes and the electrolyte [19]. The complete equivalent circuit as well as the resistance and capacitance values of the tissue model at all time points are shown in Figure 4. Fitting to the equivalent circuit was performed with the mean impedance curves obtained as explained in Section 2.4. Capacitance decreased with time in both BDL and sham-operated biopsies. On the other hand, resistance behaved differently in fibrotic and healthy tissue. While sham-operated samples presented a decrease in resistance with time, BDL ones exhibited an increase in this parameter with the evolution of fibrosis.

**Figure 5:**
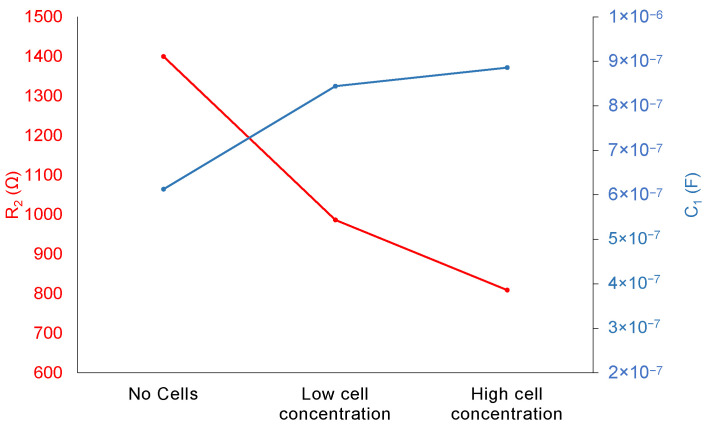
Resistance and capacitance values of the equivalent model obtained from the 3D cell culture measurements. Low and high cell concentrations correspond to $1\times 10^{5}$ cells/ml and $5\times 10^{5}$ cells/mL Hep-3B, respectively. Cells were cultured in a collagen sandwich gel at a concentration of 2 mg/mL.

It is difficult to attribute a direct biological meaning to resistance and capacitance in the tissue. In an attempt to dissect the role of these two components in the tissue impedance results obtained, we employed a 3D culture of hepatic cells in which it was possible to vary cellularity. Hep-3B cells grown in collagen-sandwiched 3D cultures served both as a validation of the model as well to determine the contribution of cell number to ECM ratio in the model’s elements. Figure 5 depicts the results obtained, which show that resistance is proportional to the ECM content and that capacitance is proportional to the cell number.

## 4. Discussion

Tissue impedance characterization relies on the fact that biological material behaves as conductors, dielectrics, or insulators depending on their composition. For this reason, impedance can provide information about the physiological state of a tissue and has been extensively studied since it is a non-invasive, non-destructive, and label-free approach [18]. Moreover, it is a quantitative and real-time technique with potentially lower costs than standard methods, rendering it more accessible for routine use. A recent study showed that impedimetric analysis of freshly dissected breast tissue had significant potential in malignancy diagnosis and, therefore, a direct clinical application [20]. In a step further towards in vivo applications, Yun et al. proposed the electrochemical impedance spectroscopy-on-a-needle for the electrical discrimination of tissues as a profiling procedure prior to biopsy [21]. Importantly, Park et al. developed an impedance-based biopsy needle for real-time sensing during the biopsy process [16]. Medical devices based on this principle are already on the market such as the ZedScan™ (Zilico). It is a handheld device that, through EIS, enhances the accuracy of the colposcopy procedure in the assessment of neoplasia, providing real-time results, hence significantly reducing the time required for diagnosis [22].

**Figure A3:**
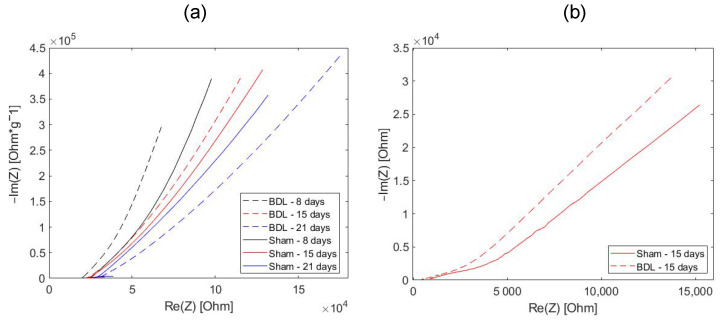
Nyquist plot of sham-operated and BDL (**a**) with the culture chamber setup and after mass normalization of the magnitude (**b**) through needle electrodes.

**Figure A4:**
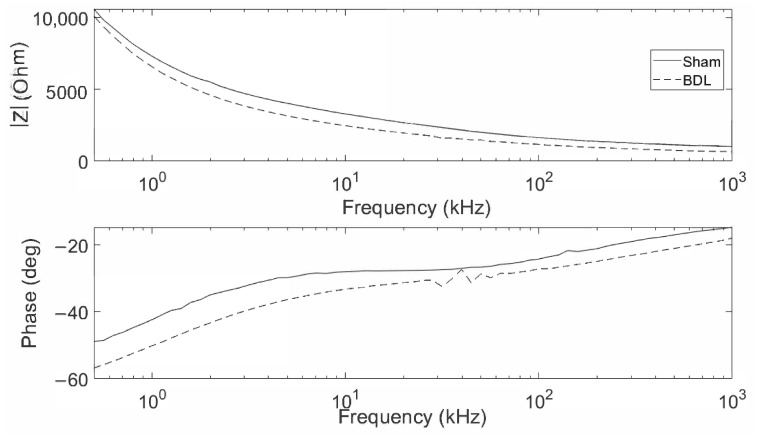
Impedance magnitude of day 15 sham-operated and BDL tissue biopsies obtained through direct insertion of needle electrodes into the tissue. The frequency was between 500 Hz and 1 MHz.

In the present work, we further assessed the diagnostic potential of EIS in liver fibrosis evolution. The studied frequency range (500 Hz–1 MHz) is mainly associated with alpha and beta dispersions, which represent intrinsic composition and properties of the tissue. Beta dispersion, specifically, is related to extracellular content and cellular membranes polarization [23]. The first set of measurements on fresh tissue biopsies in culture chambers revealed significant differences between healthy and fibrotic tissue. The subsequent measurements at different time points following BDL led to the electrical characterization of the temporal evolution of fibrosis. Culture medium and mass normalization enabled the direct comparison of impedance curves in the frequency domain (Figure 3). It was found that, at the early (8 days) and middle (15 days) fibrotic stages, healthy tissue present higher overall impedance with respect to sham livers. To confirm these findings and to discard the possibility of a bias or error introduction during the normalization step, a direct needle measurement at an intermediate fibrotic stage (15 days) was implemented (Figure A3 and Figure A4). In this setup, no culture medium was present and the electrodes were inserted directly into the tissue, thus requiring no normalization. The results obtained further support our data.

Different approaches have been proposed hitherto for modeling the dispersion relationship of soft tissue. The most used models are the Debye and Cole-Cole. Specifically, for liver tissue, Huang et al. proposed a multiscale liver bioimpedance model able to determine the impedance changes due to blood flow within the tissue [23].

In an attempt to fully characterize the system under study through physical, quantitative, and meaningful variables, impedance data were used to obtain an equivalent circuit model through a fitting procedure [19]. Tissue was represented by a single-dispersion RC model. Therefore, resistance and capacitance values were the variables under study. Capacitance showed the same behavior and very similar values in BDL and sham-operated samples for all time points. On the other hand, resistance decreased with time in sham-operated tissue in an almost linear way while BDL displayed an increasing trend (Figure 4) in accordance with the fact that ECM accumulates with time in the fibrotic liver, as shown by both molecular and histological analyses, as we recently demonstrated [13]. With the hypothesis that capacitance reflects cellular content while resistance corresponds to the overall contribution of the ECM, we assessed whether the imbalance in the hepatic tissue architecture was caused by decreased cellularity. An analysis of the 3D hepatic cell cultures confirmed the proportionality between capacitance and cell population and between resistance and ECM (Figure 5). These results were consistent with the studies of Sun-Mi et al., in which a 3D capacitance biosensor was developed for monitoring 3D-culture systems. The authors found that the increase in the number of living cells paralleled the increase in capacitance [24]. Differences in capacitance between fibrotic and non-fibrotic tissue, which account for cell number, differ at the most by 586 pF (Figure 4). The biggest difference in resistance between BDL and sham-operated measurements was found at 8 days post-surgery and was around 2 kΩ; these values became closer at 15 days and then diverged at 21 days. These results highlight that the resistance magnitude reflects the overall differences in the ECM composition at early stages of liver fibrosis. Further studies should be performed to correlate the resistance value to mechanical properties of the tissue such as stiffness, which is known to increase with the progression of fibrosis [25]. Since resistance curves of healthy and fibrotic tissue overlap, the main limitation of this approach is that resistance value alone is not sufficient for the assessment of liver fibrosis. A comparative analysis or the combined study with other variables is further required. A recent improved method for dielectric measurement of liquids proposed by Matko et al. could be explored for enhanced capacitance characterization of tissues in a culture medium or 3D cell cultures. This method, based on a capacitive-dependent quartz crystal and two quartz oscillators, provided high sensitivity and accuracy [26].

## 5. Conclusions

The main goal of the current study was to evaluate the electrical signature of hepatic tissue during early fibrogenesis through minimally destructive, label-free impedance-based analysis of biopsies. A setup made of two vertical electrodes inserted inside the culture chamber allowed for impedance-based characterization of the BDL-induced fibrotic tissue as well as for an assessment of the temporal evolution of fibrosis. Moreover, an equivalent circuit model was introduced and validated through the measurement of a 3D culture of hepatic cells seeded at different concentrations. Proportionality was observed between capacitance and cell number and between resistance and ECM. Impedance values alone, however, were not capable of discriminating between healthy and fibrotic tissue or among the fibrotic stages, thus soliciting a comparative analysis. Nevertheless, since the greatest resistance gap between healthy and fibrotic tissue was found at 8 days, this approach presents great potential for the study of fibrosis evolution at early stages. The general model and its biological interpretation may be easily extrapolated to other soft tissues. For the specific case of liver fibrosis, the findings might be exploited in a biopsy needle setup directed towards real-time EIS measurements during the biopsy process. Further studies regarding other fibrotic models and translation of the current findings to the clinics are needed.

## Data Availability

The datasets generated and analyzed during the current study are not publicly available but are available from the corresponding author upon reasonable request.

## Appendix A

Through image analysis, the dimensions of the samples were obtained in terms of the two main axes (Figure A1). Table A1 and Table A2 present the mean dimensions, standard deviation, and standard error of four measurements of each type of tissue. All of the measurements are presented in millimeters. The mean mass values of 30 tissue biopsy samples are shown in Table A3.

**Table A1:** Mean biopsies dimensions for fibrotic tissue (*n* = 4).

	Long-Axis	Short-Axis
Mean	3.23	2.22
Std. Dev	0.44	0.12
Std. Error	0.22	0.06

**Table A2:** Mean biopsies dimensions for healthy tissue (*n* = 4).

	Long-Axis	Short-Axis
Mean	3.09	2.25
Std. Dev	0.23	0.44
Std. Error	0.22	0.06

**Table A3:** Mean mass values of 30 tissue biopsy samples.

Mean Mass Values (*n* = 30)	
Mean	13.40 mg
Std. Dev	3.89 mg
Std. Error	0.71

## Appendix B

The needle electrode setup was used for direct biopsy and 3D cell culture measurements. The direct measurement without the contribution of the surrounding culture medium served as validation. On the other hand, the collagen-sandwiched 3D culture led to the determination of the biological meaning of the elements in the equivalent circuit model (Figure A2).

## Appendix C

Appealing to the widely used representation of the real and imaginary components of impedance, the Nyquist plot of the data is presented (Figure A3). Differences are observed between healthy and fibrotic tissue at all time points. The needle electrode measurements back up the initial findings for the intermediate (day 15) disease condition.

## Appendix D

For further validation purposes, a direct tissue measurement was performed using needle electrodes at the intermediate condition of 15 days after BDL or sham operation. The impedance magnitude of the BDL fibrotic tissue was lower than the sham-operated one, confirming the previous results obtained through the original setup inside the culture chambers (Figure A4).
